# Simulation and validation of hydraulic track drive systems for electrification of heavy-duty machinery

**DOI:** 10.1038/s41598-025-17449-5

**Published:** 2025-08-26

**Authors:** Hugh Coyle, Charles Young, Nicola Anderson, Lee Johnston, Robert Gilmour

**Affiliations:** 1 ATU Donegal, Port Road, Letterkenny, F92 FC93 Co. Donegal Ireland; 2 Terex Corp Terex (Omagh site), Drumquin Road, Omagh, BT78 5PN Co. Tyrone Ireland

**Keywords:** Simulink, Hydraulic track drive, Validation, Rock crusher, Mechanical engineering, Fossil fuels

## Abstract

This work explores the challenges and necessity of replacing inefficient hydraulic systems in large-scale heavy-duty off-road machinery, such as mobile rock crushers, with innovative electric solutions. A mathematical and Simulink simulation model of a rock crusher’s hydraulic track drive is developed and validated against physical test data. The model investigates the power developed by the hydraulic system during operation via simulation and is validated using results obtained from physical testing of the machine. These findings can inform the specification of an equivalent electric system, enabling similar torque delivery without the idle energy losses typical of fossil fuel systems. The simulation also supports appropriate energy storage sizing to perform required manoeuvres. The simulated system accuracy ranges from 4 to 12%, with a 20% outlier attributed to real-world inefficiencies like fluid losses and transient response delays. This level of accuracy is considered sufficient for guiding electrification efforts, ensuring the proposed electric system is neither under- nor over-designed. The insights gained from this simulation work contribute to the transition of heavy-duty machinery from hydraulic to electric powertrains, supporting the development of more energy-efficient and sustainable solutions.

## Introduction

Increasing demand for efficient, environmentally friendly and sustainable machinery necessitates a shift from currently employed hydraulic systems to equivalent electric systems. In this work, the power and energy requirements of a hydraulic track drive system for a mobile rock crusher are modelled and analysed. The objective of this simulation effort is to quantify the power, and energy demands of the existing hydraulic system to provide a basis for specifying an equivalent electric system and battery energy system. This model serves as a tool for validating the energy consumption and power delivery characteristics of the hydraulic system, which is essential to determine the requirements of a fully electrified equivalent system.

The primary challenge in this transition is the difference in power density between these systems^1–4^. Hydraulic systems and components are known for high power density which lends themselves to larger force requirements seen in heavy machinery and off-road applications^5–7^. Historically, electric systems possess a limited power density which therefore raise concerns regarding their applicability in machinery of this scale^3,8–10^. The redundancy of the hydraulic system allows the system to operate in extreme, harsh quarry conditions which is the focus of this work. The robustness afforded by the redundancy can be beneficial for the machine but it also leads to system inefficiency^11–13^. Over sizing components to facilitate this robustness and redundancy further contributes to the inefficiency of the system due to the additional component weight and fuel capacity which is underutilised in usual operations. Current hydraulic systems commonly range from power density values of 0.5 to over 22 kW/kg, fuelled by diesel engines with an energy density of approximately 12 kWh/kg^14^. The power density afforded by a Lithium-Ion battery energy storage system is currently reaching a maximum of 300 W/kg^15^, an order of magnitude less than that available from the fossil fuel based power system.

Technological advancements in the electric motor area have however improved these conditions and have arrived at a point where an equivalent electric system is possible^15–17^. Intelligent design and optimisation of electric systems can therefore mitigate the power and energy density shortfall, thus enabling a departure from the conventional hydraulic design philosophy. In contrast to the hydraulic system, the electric alternative offers an opportunity for increased efficiency and more intelligent control^13^. Electric systems can be specified according to required performance parameters, system precision, rated power and scale of the required application.

The electric design philosophy entails specifying the system to the nominal power requirement rather than designing the system to account for the worst-case scenario as in the case of the hydraulic system. Electric components however require more intensive cooling techniques due to the increased heat generated from the use of electricity as a primary energy source.

Hydraulic and electric systems present distinct thermal management requirements due to their inherent operational differences^10^. Hydraulic systems are subject to significant energy losses due to the conversion and transmission of working fluid energy to drive the mechanical parts of the propulsion components. This leads to substantial heat generation in the system which necessitates the integration of robust cooling mechanisms. Hydraulic fluids can therefore potentially overheat without effective cooling which can cause the fluid to degrade and subsequentially damage hydraulic components in the system.

In contrast, electric systems generate heat primarily through electrical losses in the power electronics components^18^. These losses include electrical resistive heating and losses in the electronics of the system. Electric configurations involve fewer moving parts but can still be subjected to considerable heat from the electricity used to power the components, especially in high power applications. The electric systems therefore require dedicated cooling solutions to ensure that each set of electronic components are sufficiently cooled to keep them within their safe and optimal thermal limits. Specific requirements for both cooling systems are dependent on factors such as the overall system design, the loading expected on the machine and the conditions of the environment and terrain expected to be traversed.

Energy recovery techniques can be incorporated to the electric system to aid the overall efficiency of the electric system via regenerative braking and intelligent energy storage management techniques^19^. This enables the system to reclaim and store energy that would be lost in the alternative hydraulic system. In addition to the improved energy efficiencies, these benefits also manifest as a reduction in component sizing and weight further improving the performance and efficiency of the system.

Hydraulic track drive systems in heavy machinery are tasked with negotiating complex and dynamic operations such as tracking, turning, and ascending inclines on variable terrain. The system must deliver high torque and power to propel large-scale tracked vehicles operating in challenging environments such as construction sites or quarries. Understanding the power and energy requirements of these systems is crucial for optimising their performance, especially when considering a shift toward electric alternatives. Conducting physical tests across all anticipated operational scenarios is however time-consuming, costly, and impractical. This necessitates a simulation approach to enables quicker iteration times.

A series of common operations and use cases that represent normal working situations for the machine are presented in Table 1.

**Table 1:** Machine use cases for onsite testing and simulation.

Direction	Orientation	Driving mode
Reverse	Flat plane	Slow
Reverse	Flat plane	Fast
Forward	Flat pane	Slow
Reverse	Incline plane	Slow
Reverse	Incline plane	Fast
Forward	Incline plane	Fast
Turning	Pivoting	Slow

The mobile rock crusher track drive hydraulic system consists of a pair of hydraulic motors that are each driven independently by a separate hydraulic pump. The hydraulic pump is driven by the output of the onboard diesel internal combustion engine of the machine. Control of the hydraulic system is achieved with the use of a pressure-compensated load-sensing system which manipulates the motor and pump characteristics using a number of directional control valves (DCV). The DCV monitors the load at the motor according to the characteristics of the working fluid and sends a signal to the hydraulic pump which varies the flow rate to meet the demand of the motor. A detailed overview of the hydraulic system employed in the relevant machine is provided in^20^. Briefly, the track terrain model concerns the linear thrust forces developed at the sprocket to propel the machine to overcome the overall resistance forces experienced at the tracks. The resistance forces analysed include rolling resistance, terrain shear, overall sinkage and the drag force attributed to the machine and traversed terrain. The governing force equation developed in^20^ is presented in Eq. 1.

1 $$\:{F}_{Thrust}=ma+\mu\:{R}_{N}+mgSin\left(\theta\:\right)+{C}_{r}{R}_{N}+\tau\:{A}_{Contact}+{k}_{c}\frac{b}{l}{z}^{n}+{k}_{\phi\:}\frac{b}{l}{z}^{n+1}+\:\frac{1}{2}\rho\:{C}_{d}A{v}^{2}$$

The hydraulic model created in this work offers a comprehensive overview of how the system performs according to the tested anticipated duty cycles, allowing for the manipulation of key parameters including driving mode, incline angles and vehicle loads. This enables the assessment of the system performance under a wide range of potential conditions, thus ensuring that the energy consumption and power requirement results calculated by the simulation model are representative of the real-world machines. The simulation model outputs include the flow and pressure data developed by the hydraulic system which can be processed to derive power and energy behaviours over time. Power is calculated based on the relationship between the hydraulic motor’s flow rate and pressure, while energy consumption is determined by integrating the power over the duration of a given operation. These insights aid the optimisation of the design of future systems, whether hydraulic or electric, by ensuring that the powertrain is tailored to the vehicle’s actual operational needs rather than being designed with a significant redundancy in mind.

The ability to model power and energy behaviours with such precision also facilitates the development of design tools for creating efficient, energy-conscious systems. A model can simulate energy consumption for various terrains which allows for the optimisation of power delivery during more energy-intensive tasks such as climbing steep grades or turning on soft ground. A simulation-driven approach therefore reduces the need for on-site testing, streamlines the design process and provides a valuable tool for engineers transitioning from hydraulic to electric systems.

This simulation method therefore represents a significant advancement in the analysis of hydraulic track drive systems employed in mobile rock crusher machines by offering a fast and efficient alternative to traditional testing methods. With the elimination of the need for physical testing in favour of simulation-based analysis, the MATLAB Simulink model serves as a powerful tool for understanding and optimising the energy performance of hydraulic track drive systems. The data generated from these simulations can be directly applied to the design of fully electrified systems, affording the ability to specify power and energy storage requirements based on verified energy consumption patterns from hydraulic counterparts.

## Methodology

The track drive system is initially modelled mathematically using expressions to describe the propulsion and frictional forces in effect at the track and terrain interactions. This is achieved using the appropriate mathematical operator blocks to represent the relevant expressions that determine the frictional and thrust forces experienced by the machine. The development of this model is provided in previous works^20^.

The hydraulic system is constructed in Matlab Simulink (Version 2024a) with the Simscape Fluids add-on providing ‘Add-on blocks’ with customisable parameters that are tailored according to the manufacturer’s specifications. The hydraulic simulation is then combined with the mathematical model to complete the simulation.

A specific set of use cases are developed for use in onsite track testing to determine the anticipated requirements of the hydraulic system and inform the design of the equivalent electric system. The devised test cases outlined in Table 1, included forward and reverse tracking, turning operations using both pivot and differential steering, and climbing or descending inclines. The ‘linear’ and ‘inclined’ driving tests are also conducted in the two speed modes of the hydraulic motor fast’ and ‘slow’. The categorisation of the speed mode is in effect is determined by the magnitude of flow that is delivered to the hydraulic motor by the pump. It is ensured that these tests adhere to the manufacturer’s recommendations so that the correct limits for the machine are employed in each case. The data obtained is then collected to validate the performance of the simulation model in terms of accuracy and function.

### Simulink model specifics

**Fig. 1:**
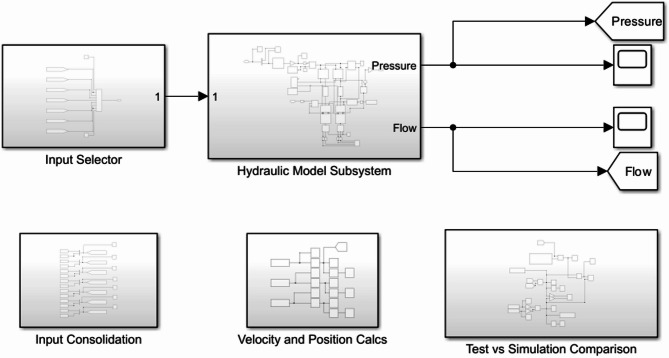
Simulink model overview.

Emphasis is placed on defining the physical hydraulic components since the model accuracy is dependent on the definition of the representative Simulink blocks. The pump and motor blocks are specified according to the actual torque, flow and speed parameters of the physical machine (Fig. 1). The input selector is used to determine which of the ideal speed profiles are used as an input to the hydraulic model subsystem. The input consolidation and velocity position calculation subsystems are used to transfer the signal variable outputs within the Matlab workspace. The comparison subsystem then transfers the required outputs to the Simulink Plot blocks to create graphical representations of the simulation outputs.

**Fig. 2:**
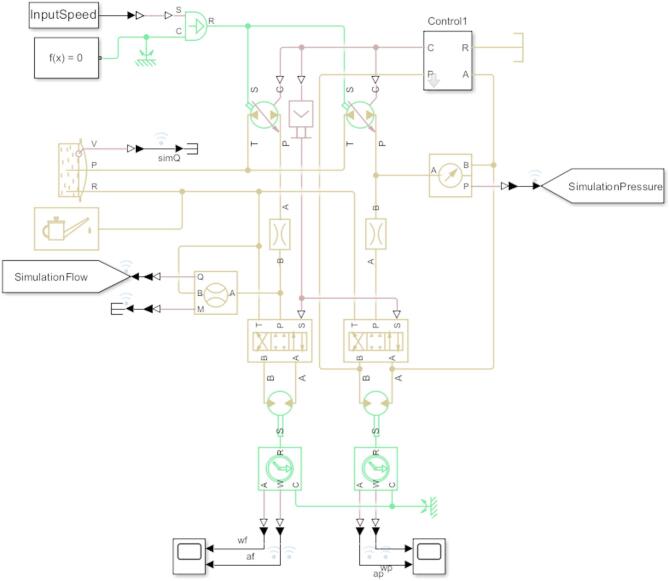
Hydraulic simulink model.

Specifics of the hydraulic Simulink subsystem are outlined in Fig. 2. Significant attributes of the system include the variable displacement hydraulic pump, the DCV and the hydraulic motor block. The system inputs comprise of a range of flow characteristics which are indicative of the onsite testing cases. These are converted into the relevant speeds via the lookup table containing the flow versus speed characteristics. The speeds are equivalent to the ICE output which drives the hydraulic pump, and they provide the input speeds for the hydraulic pump. It is assumed that the desired input speed is readily produced by the internal combustion engine output shaft which removes the need to build an extensive simulation model for that aspect of the machine. Each test case can be checked individually and is used in this manner to validate the behaviour of the model in relation to the actual machine. A sequence of speed inputs comprised of different test cases can be created using a selector block which is controlled with a Matlab variable, indicative of a desired duty cycle for the machine to perform. The load sensing and pressure compensating control is governed by the ‘Control1’ block which accounts for the required torque and loading at the hydraulic motor by varying the allowable flow accordingly. The control signal from this system is sent to the ‘Signal1’ variable and reused as the control input of the DCV before each hydraulic motor. Two sets of the components are presented since the pressure and flow of a hydraulic system in Simulink must be measured independently to ensure that the employed measurement blocks do not influence each other. The velocity, angular position and torque of the hydraulic motor is observed using the Scope block (at the bottom of Fig. 2) to ensure the motor outputs are identical between each side of the simulation. The flow and pressure variables generated by the simulation are sent to ‘GoTo’ blocks that allow the variable to be reused in other subsystems in conjunction with a ‘From’ block.

Each test case is converted into an ideal input speed for the pump and the model is validated against physical data obtained from the onsite testing. The model subsequently provides the required power plots for 30-minute drive cycles to establish the total power and energy required for the electrification of the system. The mechanical reduction gearbox transmission aspect of the machine is modelled using the gearbox block in Simulink and the track specific behaviour is expressed mathematically.

The data obtained from physical testing is first analysed in the HydroCom software of the datalogger to ensure that the plots created by the data are usable and viable. The hydraulic testing data is then exported from the datalogger into the Matlab workspace via Microsoft Excel. Matlab facilitates the creation of variables using the numerical form of the testing data, and this can be checked in Simulink using the ‘Scope’ block to ensure that the created plots compliments those presented in the HydroCom software.

## Onsite testing

The mobile rock crusher machine is comprised of two primary systems including the track drive system, which is the focus of this study, and the crushing system. The track drive system experiences a lighter duty cycle than the crushing unit due to the reduced duration in which it is in use. The operational scenarios are identified with input from the manufacturer to develop a robust testing plan which accounts for the most common manoeuvres that the machine encounters. The testing scenarios include forward and reverse tracking while also climbing and descending inclines at the specified speed modes of the machine. A pivot turning test in which one track is driven while the other remained stationary is also conducted in the half-speed driving mode. This steering method is recommended by the manufacturer instead of differential steering, which involves driving the tracks in opposite directions since this results in increased undue loading on the machine.

Onsite testing took place in a quarry with dry, firm ground conditions, which aligns with the assumptions made for the simulation model. The performance of the track drive system is measured using pressure and flow sensors fitted to both the input and output sides of the hydraulic motors for the left and right tracks. The data is logged using a HydroCOM datalogger which is attached to the sensors. Additionally, an accelerometer is attached to the machine’s chassis to record acceleration in the x, y, and z axes. Both flow and pressure data were sampled at a rate of 5 ms over a sixty-second test period, ensuring high-resolution insights into the system’s dynamic behaviour.

### Duty cycle definition

The advised upper limit for track drive operation duration is deemed to be thirty minutes which is indicative of the machine’s delivery to a new quarry site and tracking into position. This journey can involve a variety of manoeuvres outlined and they can be combined to create a duty cycle for this simulation test. A variety of tests are therefore devised to account for many common and worst-case scenarios for the machine to determine the largest potential power demand and usage for the system.

The test cases are designed to include tracking at both the half speed and full speed modes, forward and reverse tracking, skid steering to turn the machine, flat plane tracking and incline tracking. The validation of the Simulink model is attained using the data obtained from recording the hydraulic behaviour of the machine when it performs the test case operations.

## Simulation results

The Simulink model results are validated against the physical testing results obtained from the live onsite testing of the mobile rock crusher track drive system. The simulation model evaluates the hydraulic system performance by comparing the power delivery of the ‘actual’ and ‘simulated’ hydraulic motors. Model validation involves comparing simulated hydraulic power with power derived from measured pressures and flows in the actual machine. This validation step ensures that the simulation model mirrors the physical system as closely as possible to allow for meaningful comparison between both simulated and physical system outputs. Multiple statistical and validation methods are employed to achieve sufficient analysis of the accuracy of the simulation model. Error metrics and statistical calculations are applied to the results to ensure they remain within an acceptable range. Statistical correlation models are calculated using the simulation results and testing data to ensure there is agreement, consistency and a lack of significant deviation between both datasets.

System validation considers the overall hydraulic performance under varying terrain and operating conditions. Several test cases, outlined previously, provide the basis for defining the relevant terrain and operating conditions. The primary results of the Simulink model include hydraulic power outputs which can be visualised using time-series plots to allow for initial visual inspection. Additional time plots for each test case offer deeper insights into significant simulation parameters, ensuring that all aspects align with real-world machine operation. These time-series plots serve as a crucial first step in evaluating simulation accuracy before applying quantitative validation methods. The hydraulic power time-plot is the most critical analysis tool to evaluate model validation since hydraulic pressure and flow are the primary measured parameters from the onsite testing procedure.

### Simulated power results

**Fig. 3:**
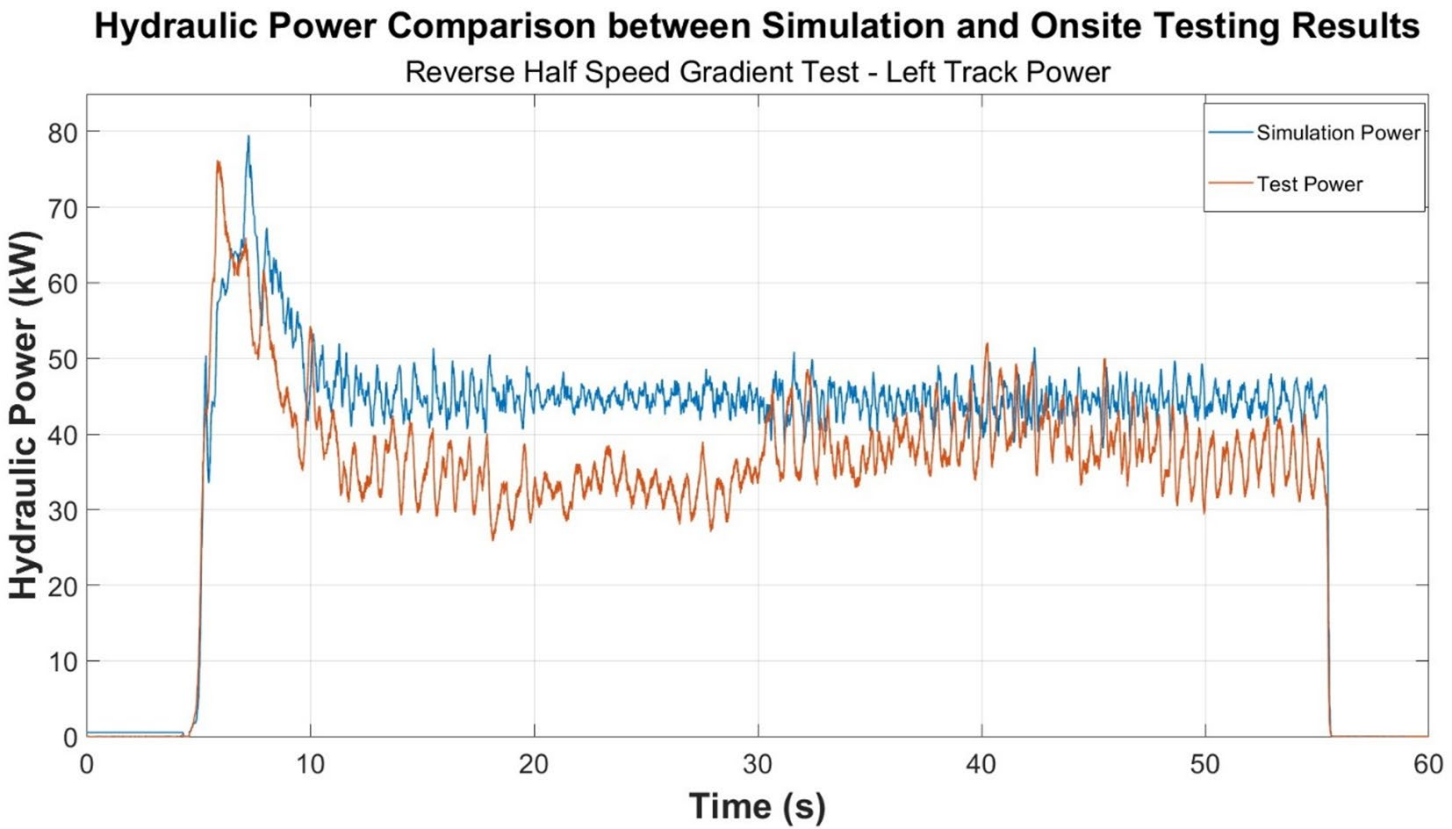
Power comparison—reverse half speed gradient test.

A comparative visualisation of the simulation and test hydraulic power generated at the left track of the track drive system for the reverse half speed gradient test case is presented in Fig. 3. At the 5 s mark approximately, both result sets experience a sharp rise due to the initialisation of the operation where both sets reach a peak of around 80 kW. The test power demonstrated more pronounced fluctuations which is an indicator of real-world system inefficiencies and response delays of the hydraulic system. The middle portion of the test period between 5 and 55 s for both the simulation and testing results approach illustrate a steadier level with the real simulated data exhibiting continued and more pronounced fluctuations. Both profiles then experienced a ‘drop off’ to 0 indicating the test cycle conclusion.

**Fig. 4:**
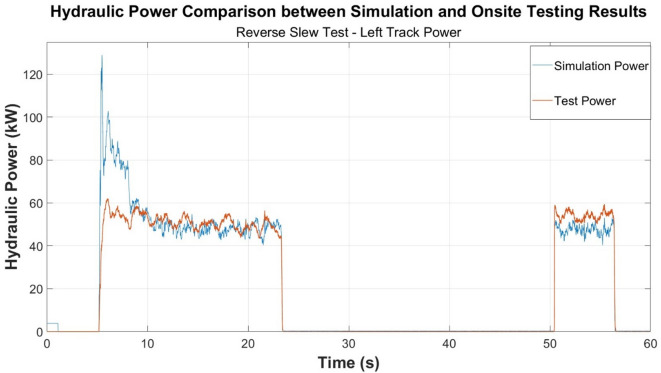
Power comparison—reverse slewing test.

**Fig. 5:**
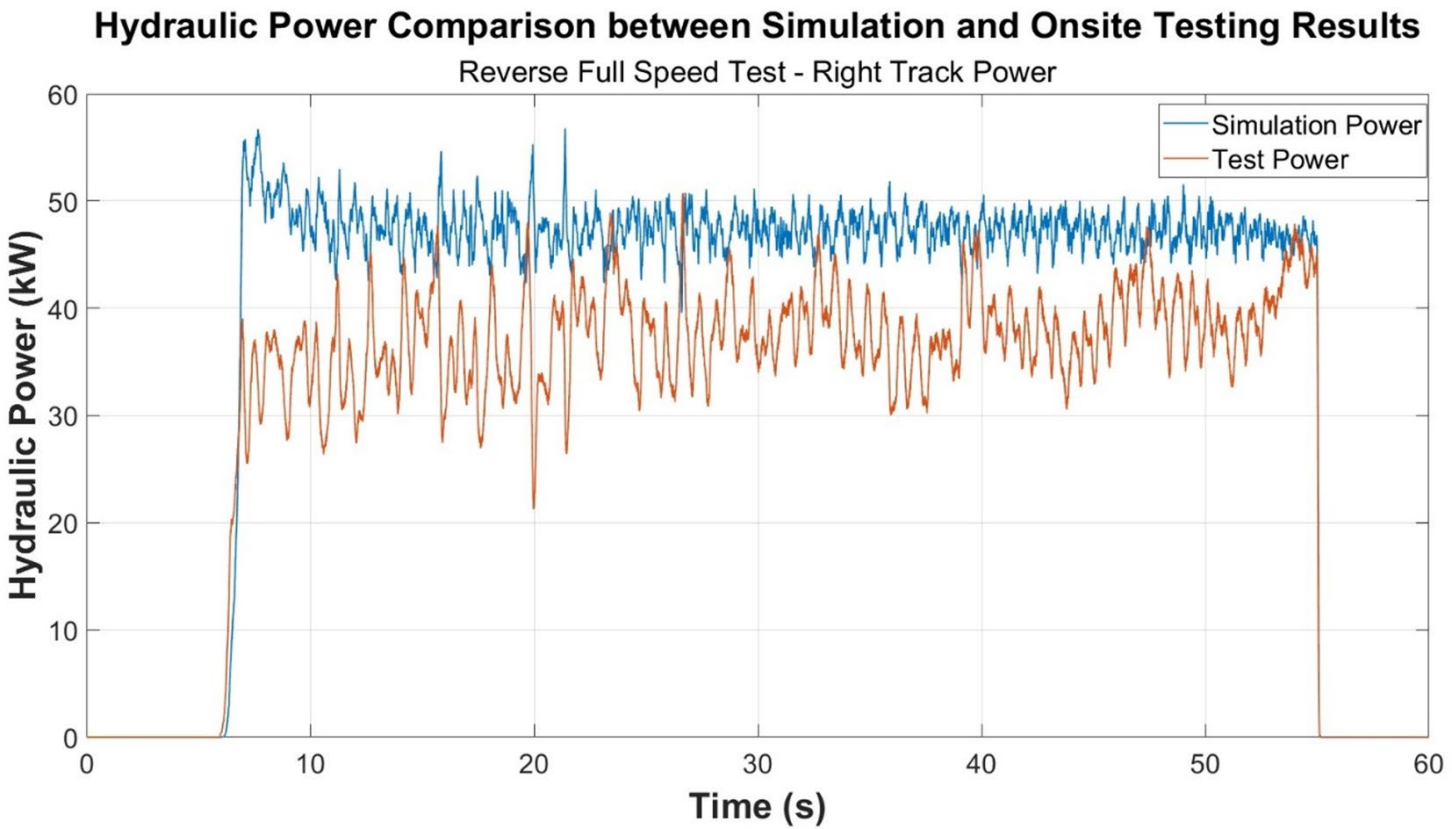
Power comparison—reverse full speed test.

Similar trends occur between each of the onsite testing and simulation test cases conducted according to the results presented in Figs. 4 and 5. Figure 4, specifically, displays the turning behaviour of the machine. The machine remains stationary between approximately 23 and 50 s hence the graph returning to zero.

A total of eight different test cases for linear tracking were examined as part of this work each representing a typical use case of the machine including operation at both driving modes including fast, slow, and flat or inclined plane tracking. Four test cases were investigated for turning operations including single track pivot turning and differential skid steering operations. Table 2 provides the primary features that are tested in each of the test cases, along with the median simulation, and the hydraulic power testing results.

**Table 2:** Median power values for simulation and test results.

Direction	Operation	Simulation median (kW)	Testing median (kW)
Forward	Flat plane	31.3	28.6
Forward	Incline plane	52.8	48.2
Reverse	Flat plane	29.8	25.2
Reverse	Incline plane	44.3	40.1
Reverse	Pivot turning	54.2	48.1
Reverse	Differential turning	53.6	49.7

Table 3 indicates the statistical error metrics applied to the simulation results in the context of the measured results for each of the test cases. The inclusion of Root Mean Square Error demonstrates the difference in the peak-to-peak datapoints of each power time-plot. The absolute errors factor the average mean values and the most median values of the power data over the 60 s timeframes. They also present the percentage error between these values for the presented test cases. The positive percentage errors are likely due to the increased attack angle of the slope encountered during that specific test causing the machine to deliver the largest power while ascending the incline. The simulation thus over compensated for the relevant terrain.

**Table 3:** Error metrics for specified test cases.

	Absolute errors		
Test case	Median (%)	Mean (%)	RMSE (kW)
Reverse full speed left	− 9.16	− 4.32	7.48
Reverse full speed right	− 11.4	− 8.37	6.67
Forward full speed gradient left	− 10.4	− 9.66	3.24
Forward full speed gradient right	− 12.6	− 7.91	4.15
Reverse full speed gradient left	− 17.2	− 12.9	6.55
Reverse full speed gradient right	− 15.9	− 20.7	8.27
Reverse half speed gradient 1 left	− 14.2	− 12.2	8.91
Reverse half speed gradient 1 right	− 10.7	− 7.20	7.16
Reverse half speed gradient 2 left	9.7	7.27	5.11
Reverse half speed gradient 2 right	10.9	6.85	4.54

### Simulated energy consumption results

Energy consumption behaviour throughout the testing duration can be analysed by comparing the simulated and onsite testing power data. This is achieved by applying numerical integration methods within Simulink to compute energy from the power signals of different test cases. Since energy is derived from power, the trends and relationships observed in the power data are reflected in the energy consumption results.

The Simulink Integrator block performs numerical integration on the input power signal over time, providing both total and cumulative energy consumption throughout the simulation. While this integration can also be performed in the MATLAB workspace, implementing it within Simulink allows for real-time computation. This enables efficient analysis by seamlessly switching between test cases for analysis.

**Fig. 6:**
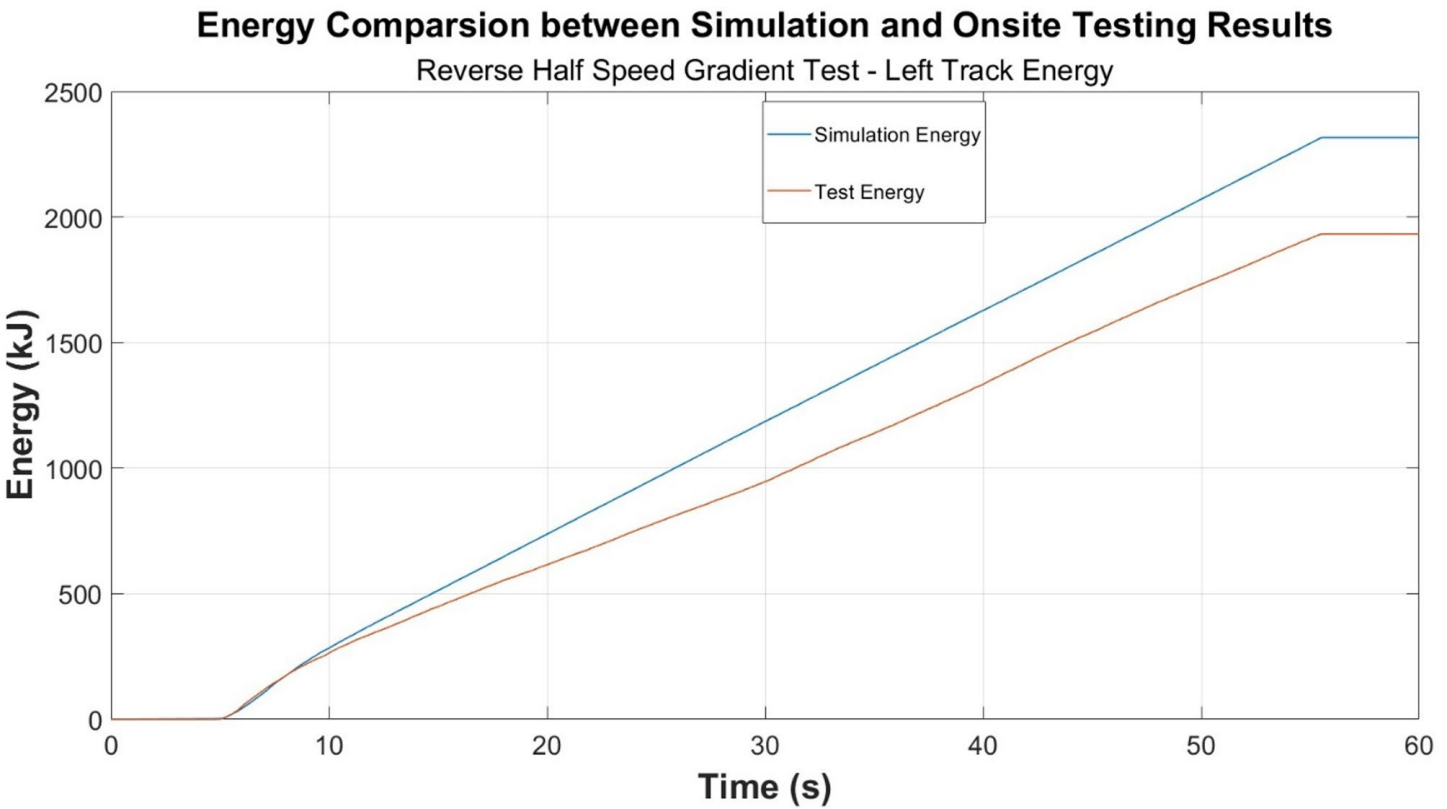
Energy comparison—reverse half speed gradient test.

**Fig. 7:**
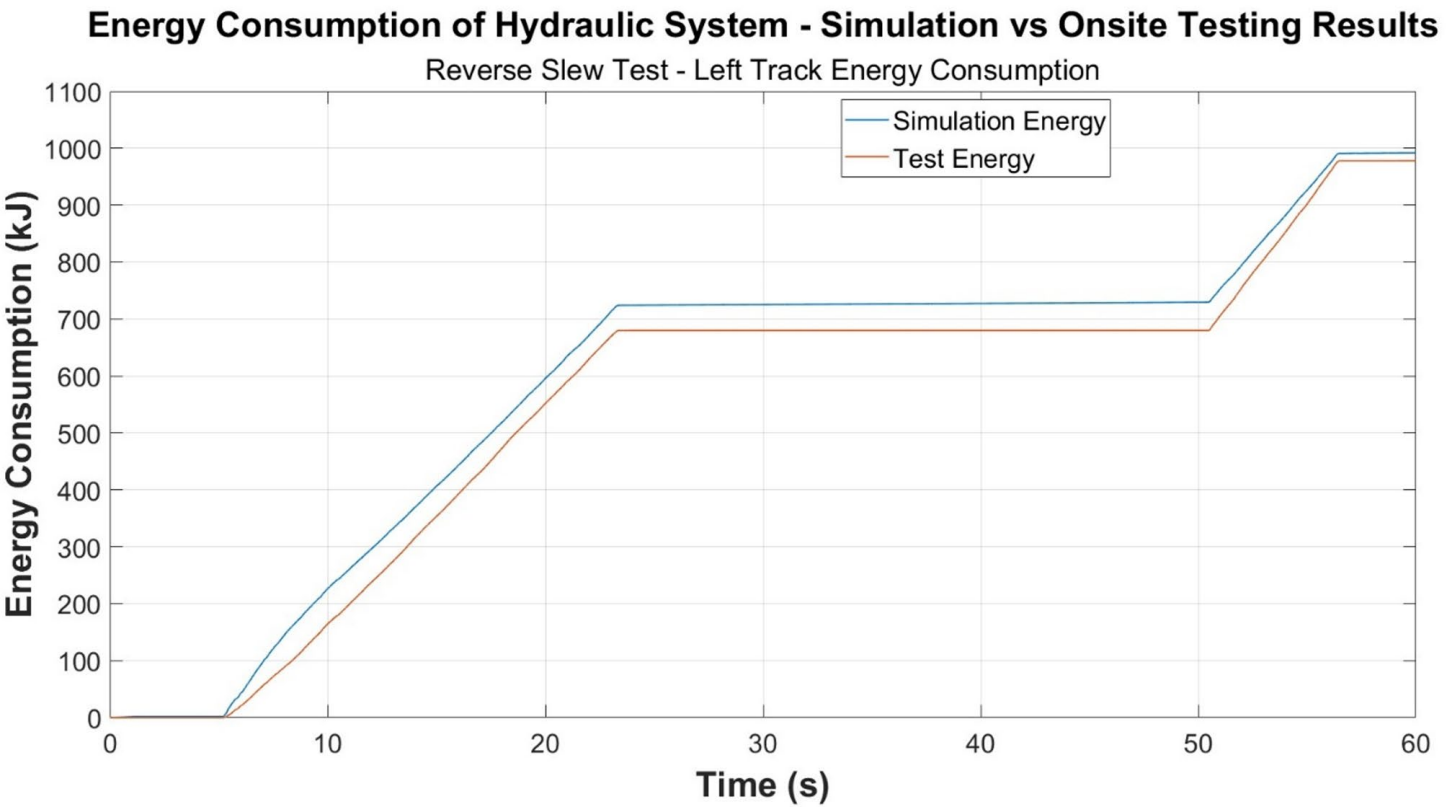
Energy comparison—reverse slew test.

**Fig. 8:**
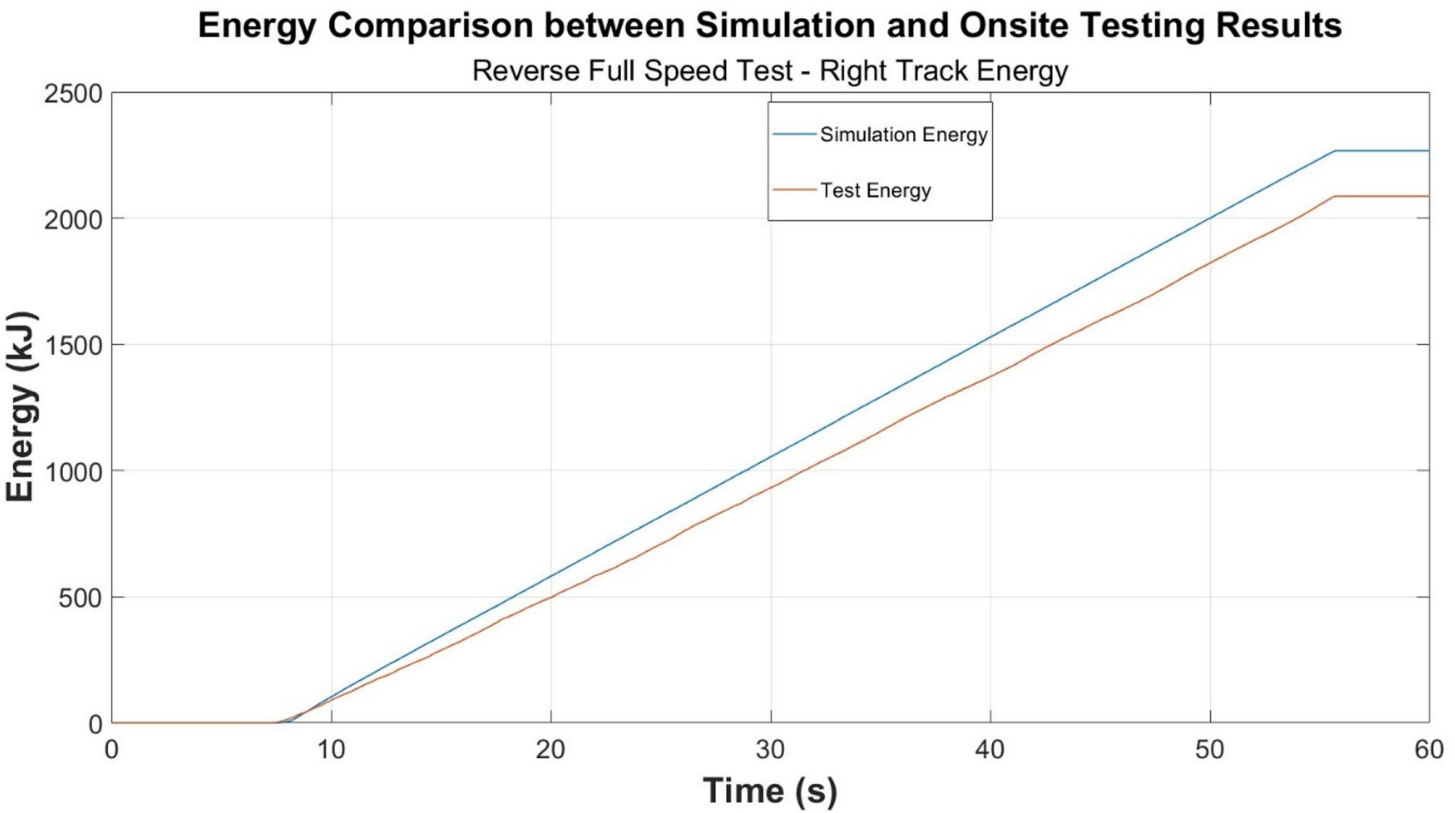
Energy comparison—reverse full speed test.

Energy consumption time-plots are developed and presented in Figs. 6, 7 and 8, and the previously explored test cases for power are presented in Figs. 3, 4 and 5.

The total energy consumption for each test case are presented in Table 4. Each drive mode and plane orientation are selected along with both pivot and differential turning operations. The total values obtained from the simulation and testing are also presented. Linear tracking operations are conducted over 60 s, while both of the turning operations are conducted over 45 s.

**Table 4:** Energy consumption results.

Direction	Operation	Simulation consumption (MJ)	Testing consumption (MJ)
Forward	Flat plane	2.419	2.239
Forward	Incline plane	1.106	0.892
Reverse	Flat plane	2.193	2.143
Reverse	Incline plane	3.164	3.827
Reverse	Pivot turning	0.513	0.415
Reverse	Differential turning	0.261	0.187

## Discussion

The accuracy of the track drive simulation model is influenced by several relevant factors, with the most significant being the fundamental assumptions made during the modelling process. It is apparent that the highly idealised assumptions made to create this model influence its direct accuracy with the physical testing. The deviation is however consistent and quantifiable so the model results can be interrogated sufficiently to show validation. These assumptions affect the model’s ability to simulate the performance of the real-world hydraulic system and directly influences its predictive accuracy. One of the most critical assumptions is that the model aims to represent an ideal operation of the hydraulic system, which does not account for real-world inefficiencies such as fluid leakage, mechanical wear, and thermal variations. While this approach ensures model stability and computational efficiency, it inherently results in higher simulated power values compared to the measurements obtained from the onsite testing data. An interrogation of these discrepancies is required to validate the model, and as such this is the focus of the subsequent section.

### Model assumptions and idealised conditions

The simulation model operates under idealised conditions that may not fully account for real-world losses such as hydraulic inefficiencies, friction, and thermal effects. For instance, the hydraulic components in the physical system experience fluid leakage, temperature variations, and mechanical wear. Whilst these reduce the actual power output in real life their significance is limited in relation to the overall scale of the machine, and the challenge associated with their representation renders their consideration redundant. In contrast, the Simulink model assumes optimal performance which leads to higher predicted power values over those observed during the onsite testing of the machine.

Environmental conditions, including slight changes in terrain and temperature fluctuations cause variations in hydraulic fluid viscosity which can significantly affect machine performance. These factors are challenging to simulate with high fidelity and will also result in discrepancies between predicted and measured power values. For this application of simulation of the hydraulic system, the achieved accuracy is satisfactory to guide the future work of electrification by providing the desired insights into the energy consumption of the system.

### Physical system and simulation model instability

Consistent spikes are observed in each presented test at the initiation of each test case. This is evident in both the simulated and measured results. At the initiation of operations, the hydraulic pump and motors experience abrupt changes in pressure and flow rates due to the system ramping up to the required force output. This transient behaviour can lead to momentary overshoots in force as the system compensates for the inertial resistance of the machine’s mass and the track-terrain interface. This initial motion can cause a rapid redistribution of forces along the machine’s tracks, particularly with frictional forces as explored in^18^.

Dynamic aspects of the system including transient behaviours and interactions between hydraulic components have also been simplified in the simulation model. For example, the simulation operates under the assumption that there is instantaneous pressure build-up and release in the ideal simulated hydraulic system, whereas for the real-world system, response times that delay peak power output exist, but these are not indicative in the testing data obtained. These simplifications, while necessary to ensure model stability and computational efficiency, can lead to the slight overestimation of power consumption observed in the simulation and testing comparisons.

### Sensor responsiveness and sample time

The sensor sample rate is set to 5ms leading to ensure that a thorough representation of the transition rate of the fluid within the system is obtained and ultimately provide an accurate measurement of the hydraulic performance of the system. The sample time of the Simulink model cannot be fixed to this same sample time due to the behaviour of the Simscape blocks used to model the system. Specifically, locking the simulation time to 5ms would increase the overall computation time for the model, so a variable step size solver is selected to vary the sample time according to the relevant Simulink block.

The physical test data is reliant on the accuracy and responsiveness of sensors used during the onsite testing of the machine. Potential delays or inaccuracies in the sensors, particularly during transient events, could result in lower recorded power values. Additionally, data acquisition systems could have introduced minor latency or noise into the measurements, thus contributing to the observed differences between simulation and physical data.

### Energy consumption analysis

Energy consumption across each test case is evaluated using the Simulink Integrator block which implements numerical integration methods to obtain the energy results from both the simulation and testing data. The results present a direct correlation between energy consumption, the power delivered during testing and the simulation model, since energy is strictly dependent on the power demand over time. A key observation that can be taken from the presented results is that incline tracking operations consistently exhibit higher energy consumption and power demand compared to linear tracking on a flat plane. This can be attributed to the additional resistive frictional forces acting on the system and the increased gravitational load induced by the inclined traversal.

The additional requirements for energy and power can continue to increase depending on the slope of the incline encountered. Increasing the incline angle causes a knock-on effect to increase the torque which leads to an increased draw in power. All of this incurs a following increase in the energy consumption of the system over the duration of the manoeuvre. The turning operations are analysed over a shorter time period whereas the machine only conducts the manoeuvre for approximately 15 s. Over this duration, the power demand of the turning operation almost matches that of the incline plane traversal operation. Similarly, in the case of the incline operation, the turning manoeuvre experienced additional resistive forces that are not present in the linear flat plane tracking operation.

Although the test duration for the turning operation was shorter than the linear tracking tests, it can be seen that in comparison the turning operation would undergo similar power demand to the incline tracking operation. Converting this power demand over the duration to energy consumption, demonstrates that the slope of the actual profile of the slewing energy consumption is similar to both linear tests. An issue arises however when the energy per distance traversed is analysed since the turning operation involves minimal traversal rather the machine pivoting in place to achieve the turning motion. This highlights the significant inefficiency of the turning operation in relation to the linear tracking operations. This increased energy requirement can be attributed to the slip forces and lateral effects experienced by the machine when turning which necessitates a greater power demand to complete the turning manoeuvre.

### Implications for electrified solutions based on energy analysis

The total energy consumption of the hydraulic system serves as a fundamental baseline to determine the energy requirements of an equivalent electrified system that replaces the hydraulic system. The electrified solution is expected to both replicate and improve upon the operational performance of the hydraulic counterpart. Analysis of the hydraulic power demand over various operating conditions of the machine provides crucial insights for the designing of an electric alternative. Comparison between hydraulic and electric systems ensures that the electric system is neither over-designed nor under-designed. Over-designing the system would require incorporating an excessive redundancy to afford the system the ability to negotiate any potential requirement. This approach could result in excessive costing and overall weight gain. Under-designing could lead to system performance limitation preventing the machine from completing basic requirements. Worst case energy consumption for the tested machine and validated results for the 60 s test duration amounted to 3.8 MJ. The electric system is posited to operate for only a half hour duty cycle. Again, taking worst case approximations, the energy consumption can be extrapolated to 114 MJ. An expected energy storage system for this energy usage over the provided time with the necessary adjustments for peak charging characteristics is approximately 40 kWh.

Energy consumption in hydraulic systems is influenced by factors including fluid leakage, response time of the motor to reach desired torque, overall hydraulic system performance and the efficiency of the mechanical components. Inefficiencies introduced by these factors can lead to higher energy losses due to friction, pressure drops and the inherent energy dissipation of hydraulic components. Electrified systems have no equivalent to fluid leakage, but inefficiencies can still arise from electrical and thermal losses in the inverter, motor, and battery system. Similarly, electrified systems involving an electric motor, lack the response time taken to build the desired torque as seen in hydraulic systems. This is due to the constraint provided by the fluid dynamics and valve responses inherent in hydraulics. Electric motors can deliver near-instantaneous torque as a result of the direct electromagnetic interaction between the motor components. The major challenge presented by the electric motor is the management of the thermal aspects of the power electronic components which has an influence on the efficiency and energy consumption of the electric equivalent solution. The battery is also subjected to thermal conditions unlike an ICE system. The battery storage system can perform with varying efficiency depending on the thermal use conditions. Preheating the battery is employed in passenger EVs for enhanced charging and cooled when discharging to prevent thermal degradation of the battery chemistry. This is extremely important here due to the increased torque demands by the machine leading to higher discharge rates which increase the potential cooling required to keep the battery at the desired safe and efficient operating temperature.

Battery capacity sizing can be linked to the energy consumption of the hydraulic system since both systems require similar operating demands. The characteristics of the hydraulic system can serve as a reference to base the minimal requirements for capacity of a potential battery solution. Sufficient energy must be available and provided by the battery solution to enable the electrified solution to complete the equivalent use operations, while also factoring in enough energy for emergency use cases for prolonged operation cases. A compromise must be made to ensure that enough energy is available without a significant unmanageable increase in vehicle weight, component costs or packaging constraints.

Sizing of the electric motor should be based on the hydraulic power observed from the testing and simulation of the current system. The selected component should be comparable to the hydraulic motor counterpart but must be specifically optimised for the nominal power requirements of the system, rather than peak loads experienced. Hydraulic actuators used alongside hydraulic motors can handle high transient loads efficiently by utilising fluid accumulators, which can store, and release energy as needed. Electric motors must be designed to handle both steady-state and transient power demands without excessive oversizing since larger motors result in higher mass with more complex thermal management and control requirements which can lead to higher energy consumptions.

## Conclusion

This work presents a validated simulation model of the hydraulic track drive system of a mobile rock crusher which accurately determines the power demands and energy consumption experienced by the system during common operating conditions. Analysis of the performance of the simulation model is conducted to determine the robustness and accuracy of the model in comparison to the real-world performance of the system. The model demonstrates high accuracy, with mean percentage errors ranging from 4 to 12%, and a single outlier at 20%, validating its reliability for informing the transition to an electric equivalent.

Simulated power demands and energy consumptions are validated against the onsite testing data which provides critical benchmarks to compare and specify an electrified track drive solution. These factors ensure that correct motors are selected, sufficient battery capacity is available, and sufficient power control can be implemented to ensure the effectiveness of the system. The robustness of the control system for an electric solution is necessary to avoid conforming to the over specified design philosophy of the current hydraulic system. Increased thermal management is also necessary due to the significantly increased higher heat generated by the electric components. Both of these factors are required to ensure the electric solution is comparable or superior to the performance of the current system.

This validated model serves as an important tool to guide the electrification of mobile rock crusher track drive systems. This transition aims to reduce dependency on the fossil fuel-driven hydraulics. The insights derived from the validation of the simulation model can support the design of more energy-efficient, sustainable, and high-performance electric track drive system for mobile rock crushers, ensuring that they can also be employed in similar heavy-duty machines and applications going forward.

## Data Availability

The data can be made available upon request. Contact corresponding author with any queries.

## Abbreviations

DCV: Directional Control Valve
$$\:{F}_{Thrust}$$: Thrust Force (N)
$$\:m$$: Mass (kg)
$$\:a$$: Acceleration (m/s^2^)
$\:\mu\:$: Coefficient of friction
$\:{R}_{N}$: Normal Reaction Force (N)
$\:g$: Acceleration due to Gravity (m/s^2^)
$\:\theta\:$: Angle of incline (°)
$\:{C}_{r}$: Coefficient of Rolling Resistance
$\:\tau\:$: Terrain Shear Force (N)
$\:{A}_{Contact}$: Track Area of Contact (m^2^)
$$\:{k}_{c}$$: Terrain Stiffness
$$\:b$$: Track Width (m)
$$\:l$$: Track Length (m)
$$\:{z}^{n}$$: Sinkage depth (m)
$$\:{k}_{\phi\:}$$: Terrain parameter
$\:\rho\:$: Density of Fluid
$$\:{C}_{d}$$: Drag Coefficient
$\:A$: Cross Sectional Area in Contact with Fluid (m^2^)
$$\:v$$: Velocity (m/s)
